# Partial Oxidation of Methanol on Gold: How Selectivity Is Steered by Low-Coordinated Sites

**DOI:** 10.1021/acscatal.3c04578

**Published:** 2024-05-06

**Authors:** Salma Eltayeb, Lenard L. Carroll, Lukas Dippel, Mersad Mostaghimi, Wiebke Riedel, Lyudmila V. Moskaleva, Thomas Risse

**Affiliations:** † Institut für Chemie und Biochemie, Freie Universität Berlin, Arnimallee 22, 14195 Berlin, Germany; ‡ Department of Chemistry, Faculty of Natural and Agricultural Sciences, University of the Free State, P.O. Box 339, Bloemfontein 9300, South Africa; § Institute of Nanotechnology, Karlsruhe Institute of Technology (KIT), P.O. Box 3640, 76021 Karlsruhe, Germany

**Keywords:** gold, methanol, oxidation, Au(111), Au(332), low-coordinated sites

## Abstract

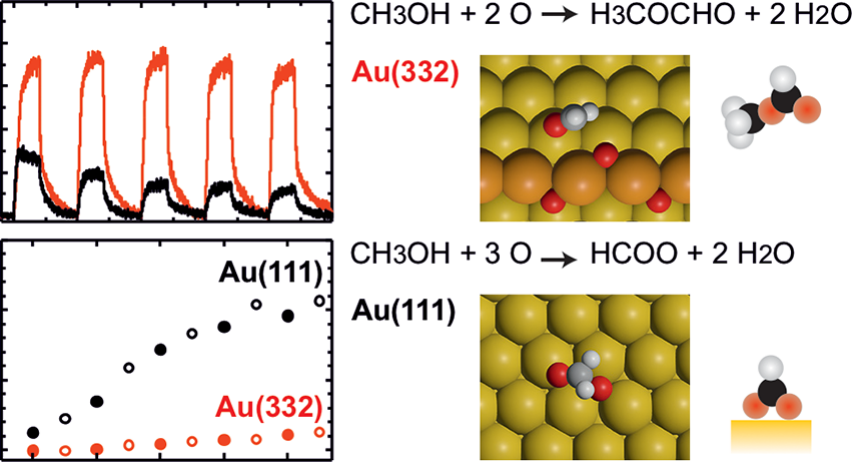

Partial methanol oxidation proceeds with high selectivity to methyl formate (MeFo) on nanoporous gold (npAu) catalysts. As low-coordinated sites on npAu were suggested to affect the selectivity, we experimentally investigated their role in the isothermal selectivity for flat Au(111) and stepped Au(332) model surfaces using a molecular beam approach under well-defined conditions. Direct comparison shows that steps enhance desired MeFo formation and lower undesired overoxidation. DFT calculations reveal differences in oxygen distribution that enhance the barriers to overoxidation at steps. Thus, these results provide an atomic-level understanding of factors controlling the complex reaction network on gold catalysts, such as npAu.

Methyl formate (MeFo) is an important precursor for numerous bulk chemicals, including formic acid and dimethylformamide, a potential fuel substitute (or additive), and also a prospective hydrogen energy carrier.^1−5^ Currently, MeFo is mainly produced by the reaction of methanol with CO over alkali methoxide catalysts requiring dry and CO_2_-free reactant feeds.^2,3,6^ Hence, alternative routes are being explored, such as methanol dehydrogenation or partial oxidation, with the latter being thermodynamically favored.^1,2^ Nanoporous gold (npAu) catalysts show high activity and selectivity toward MeFo in the aerobic partial oxidation of methanol at low temperatures (below 100 °C).^7^ The residual less noble metal (typically Ag) in the nanometer sized ligaments of npAu, which remains after preparation, is crucial for the catalytic activity, as it enables the activation of molecular oxygen.^7,8^ However, increased amounts of residual Ag and high oxygen partial pressures in the reactant feed also promote unwanted overoxidation of methanol to CO_2_ (and water).^7^ Mechanistically, different oxygen species present on npAu were shown to influence the selectivity of the catalyst, which is in line with experimental and theoretical findings on model systems.^9−20^ Structure-wise, the npAu ligaments exhibit a mixture of low-index terraces and a significant number of low-coordinated sites,^21^ which have been suggested to affect selectivity in methanol oxidation on npAu, too.^15,16,22,23^

To gain insights into the underlying reaction mechanisms at the atomic level for the aerobic methanol oxidation on npAu, model studies on gold surfaces under well-defined ultrahigh vacuum (UHV) conditions have been successfully employed.^16,17,24^ In these UHV studies, the use of activated oxygen, e.g., atomic oxygen or ozone, is required, as molecular oxygen does not dissociate on gold under UHV conditions.^25,26^ Based on temperature-programmed reaction (TPR) studies on Au(111) and theoretical calculations, a reaction mechanism was proposed (Figure 1).^17,24,27^ In brief: first, the reaction of methanol with adsorbed oxygen species results in methoxy species (1) and subsequent β-hydride elimination leads to formaldehyde (2), which is the rate-limiting step in MeFo formation in the presence of activated oxygen.^17,24,27^ The desired MeFo is formed by a coupling reaction of formaldehyde with adsorbed methoxy and subsequent hydrogen abstraction from the resulting intermediate by adsorbed oxygen species (3, blue path). However, there are competing pathways to the coupling of formaldehyde: it can be further oxidized first to formate that finally leads to undesired total oxidation (5a–5c, red path). Alternatively, it may desorb (4, black path). Under single-collision conditions, as in molecular beam (MB) experiments on Au single-crystal surfaces, the latter process reduces the selectivity to MeFo, while for ambient pressure catalysis on npAu, subsequent collisions of desorbing formaldehyde along the catalyst bed can result in MeFo formation or overoxidation. While we anticipate that the competing reaction channels are influenced by both the surface morphology and the nature of the oxygen species associated with different surface sites, a detailed understanding of how these factors affect the selectivity is still missing.

**Figure 1:**
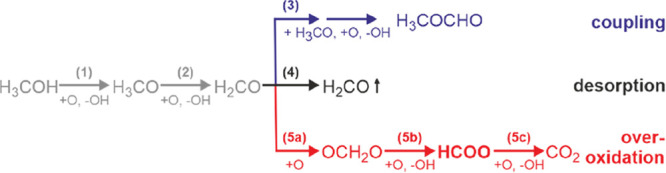
Reaction mechanism for methanol oxidation on gold.

In this study, we compare model catalysts with different surface structures: a flat Au(111) surface and a stepped Au(332) counterpart composed of six-atom-wide (111) terraces separated by monoatomic close-packed steps. We used pulsed MB measurements under well-defined single-collision conditions to study the isothermal reactivity. As an advantage over more commonly used TPR techniques, this method allows us to investigate the isothermal kinetics of both MeFo formation and overoxidation. We also use DFT-based computations to gain insight into the reaction energetics on different surfaces. We show that a higher MeFo selectivity of Au(332) could be connected to the formation of extended Au–O chains along steps because O atoms within such chains are shown to exhibit a different reactivity as compared to individual O atoms. The results improve our understanding of the complex reaction network that determines the selectivity of gold catalysts, such as npAu or Au nanoparticles, and also highlight the importance of such comparative studies for other structure-sensitive catalytic reactions.

Figure 2a reports the amount of MeFo produced on Au(111) (black) and Au(332) (red) in isothermal pulsed MB experiments at 230 K using a 10-fold excess of methanol flux compared to the flux of atomic and thus activated oxygen during the pulses (see the SI for details). The initial formation rate of the desired partial oxidation product MeFo is clearly lower (by a factor of ∼2.5) on Au(111) than on stepped Au(332), showing that MeFo is formed more easily on a stepped surface with abundant low-coordinated atoms. Along the pulse sequence, the MeFo formation decreases significantly for Au(111), while little deactivation is observed on Au(332). Concomitantly, the difference in MeFo formation of the two surfaces increases, being ∼5.5 times higher for Au(332) at the end of the pulse sequence. Accordingly, the MeFo selectivity with respect to the provided oxygen flux decreases (see the SI for details). As discussed in Figure 1, the desorption of formaldehyde (and methanol) will reduce the selectivity to MeFo under the single-collision conditions of these experiments. In turn, a higher adsorption energy, as expected for low-coordinated sites, leads to higher transient surface concentrations (the reaction temperature is above the desorption temperature for both formaldehyde and methanol), increasing the possibility for the coupling reaction. Oxygen atoms preferentially adsorb at low-coordinated sites, e.g., step sites of Au surfaces.^15,20^ While this effect may also enhance the rate of MeFo formation, it has been shown that high local oxygen concentrations can cause overoxidation, which lowers the MeFo selectivity.^17,23^ At the reaction temperature used in these experiments, overoxidation (Figure 1, red path) leads to formate accumulation on the surface that poisons the surface for MeFo formation.^16,17^ To this end, we used in situ IR spectroscopy (IRAS) to characterize surface-bound species.

**Figure 2:**
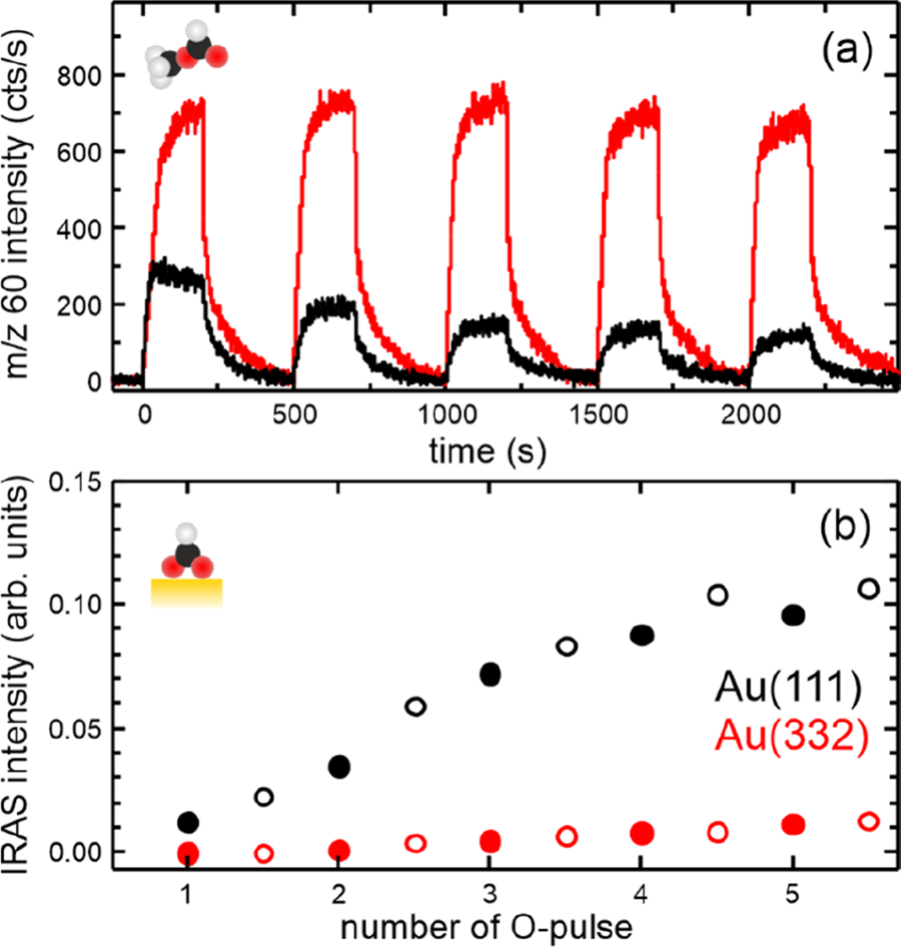
Isothermal, pulsed MB measurements on Au(111) (black) and Au(332) (red) at 230 K with a continuous methanol supply and a pulsed flux of atomic oxygen. (a) MeFo formation rate measured by mass spectrometry (*m*/*z* = 60). (b) Formate accumulation given as an (integrated) ν_s_(OCO)-IRAS-intensity around 1330 cm^–1^.

The integrated IRAS intensity of a band around 1330 cm^–1^ assigned to the ν_s_(OCO) vibration of formate (Figure 2b and Figure S1) increases along the pulse sequence for both Au(111) and Au(332). However, the signal intensity for Au(111) is much larger than that on Au(332) (factor of ∼8 after pulse 5) indicating a much higher formate accumulation on Au(111), which correlates with the stronger decrease in the MeFo formation rate on Au(111). One should be aware that oxygen atoms not only cause the formation of adsorbed formate but also cause its decomposition. Therefore, a higher formate accumulation on Au(111) can be either due to a faster formation or a slower decomposition.^16,28^ To distinguish between the two possibilities, the formate decomposition rate by oxygen atoms was investigated independently (Figure S2). These experiments show comparable formate decomposition rates for both surfaces in the pulsed isothermal MB experiments. Hence, the higher formate accumulation on Au(111) than on Au(332) must be due to faster formate formation. These experimental results show that low-coordinated step sites not only enhance the formation of the partial oxidation product MeFo but also suppress undesired overoxidation.

To better understand the differences in overoxidation observed experimentally, we performed DFT calculations (for details, see the SI) for flat Au(111) and stepped Au(221). Like Au(332), Au(221) has (111) terraces and close-packed straight steps but a smaller terrace width (three atoms wide) resulting in a smaller unit cell, thus reducing calculation times. We calculated the reaction energies for overoxidation (Figure 3) referenced to the reactants at infinite distance (indicated by a ∞ symbol in Figure 3), which allows for a rigorous comparison of the surfaces but corresponds to a low-coverage situation so that the reaction energies for coadsorbed reactants are also given. According to the accepted reaction mechanism (Figure 1, red path), overoxidation involves an oxidative attack of formaldehyde by oxygen to yield an oxidized OCH_2_O intermediate (5a), which then reacts with oxygen to yield formate (5b). Subsequently, formate reacts with oxygen to produce CO_2_ (5c). First, we compared the reaction of formaldehyde with atomic oxygen on Au(111) (blue trace) and Au(221) (orange trace, Figures 3b and 3c). Comparable reaction energies and activation barriers are found for both surfaces: the reaction of formaldehyde with atomic oxygen to form OCH_2_O is exothermic with nearly no barrier. The formation of formate is also exothermic and has only a moderate barrier for both surfaces. The subsequent formation of CO_2_ exhibits similarly high barriers on both surfaces and is found to be strongly exothermic. Note that the results for Au(111) agree well with a recent study by Réocreux et al.^29^ While the significant barriers to CO_2_ formation are consistent with the observed formate stability, i.e., slow decomposition combined with faster formation resulting in formate accumulation, comparable formate formation rates are expected from the calculations for both surfaces. This is in contrast to the experiments showing much slower overoxidation for stepped Au(332). Therefore, reactions with atomic oxygen alone cannot explain the observed differences in the overoxidation for both surfaces.

**Figure 3:**
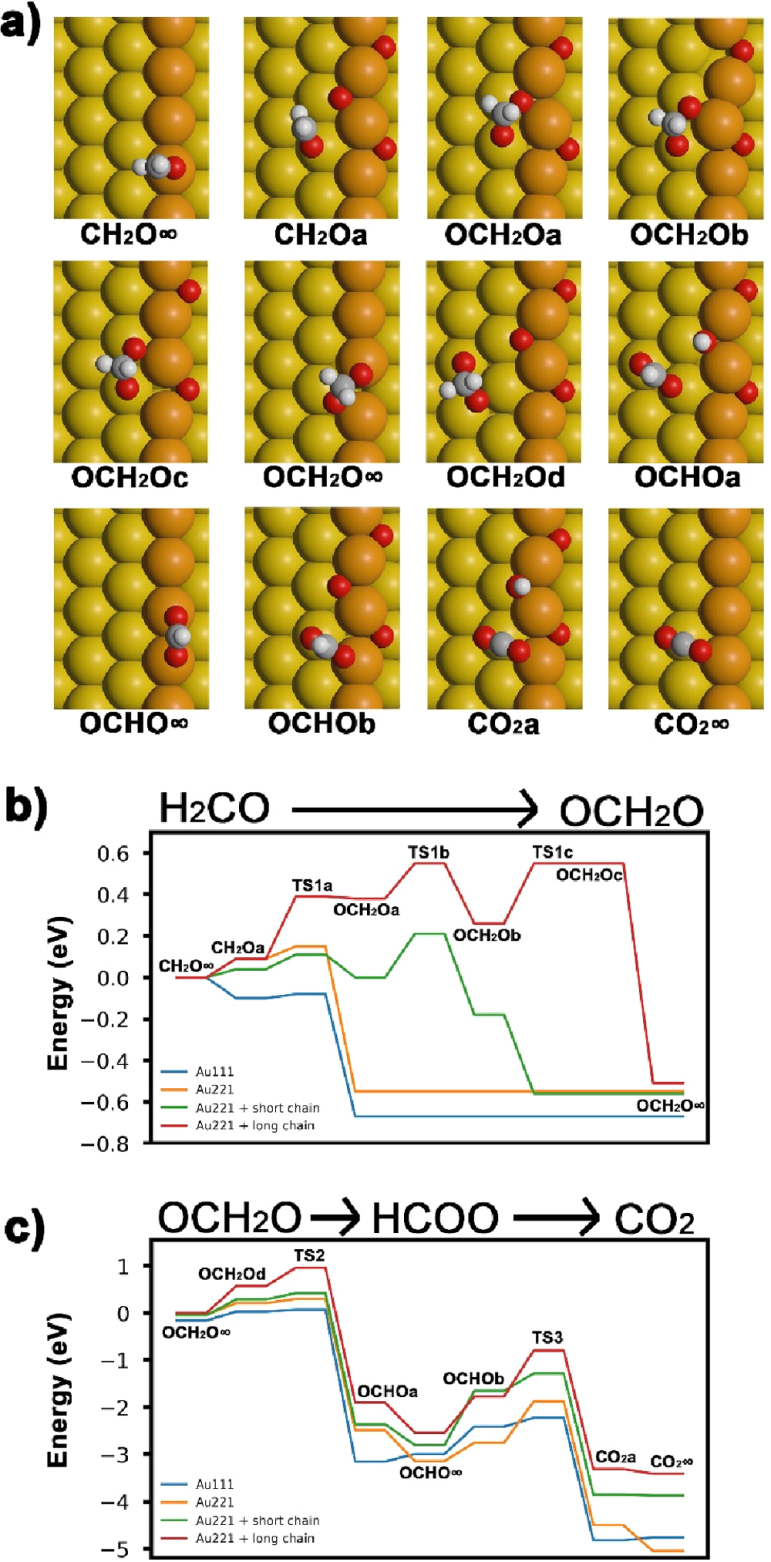
Computed reaction pathways for the overoxidation of formaldehyde. (a) Intermediate structures on the Au(221) surface. Letters next to chemical names indicate different coadsorption states of CH_2_O, OCH_2_O, OCHO, and CO_2_. The infinity symbol designates the limit of low coverage (= no coadsorbates). (b and c) Reaction energy diagrams via OCH_2_O (b) and HCOO toward CO_2_ (c) for atomic oxygen as the oxidant on flat Au(111) (blue) and stepped Au(221) (orange) as well as for short (O–Au–O, green) and long (O–Au–O–Au–O, red) Au–O chains as oxidants on Au(221).

Previous calculations have shown that oxygen atoms tend to form Au–O chain-like structures at low-coordinated sites on kinked and stepped Au surfaces,^15,20,30,31^ and similar Au–O–Au–O–Au structures have been evidenced by Raman spectroscopy at low-coordinated sites of Au nanoparticles.^32^ Experimentally, TPR and isothermal MB studies have shown that the chemical properties of aggregated oxygen species (mostly subsumed as AuO_*x*_ phases) depend on the properties of these phases, such as their size, and differ from those of adsorbed oxygen atoms.^16,22,30,31,33^ Therefore, we computed the overoxidation energetics for two model “Au–O chains” on stepped Au(221): first, a short O–Au–O–chain (green trace, Figures 3b and 3c), as a model for terminal oxygens at the end of short or longer chains, and second, for the reaction on the central oxygen of a longer chain (red trace, Figures 3b and 3c) to address the reactivity of oxygen within longer chains. For the terminal oxygen atoms, the barriers to the formation of the OCH_2_O and HCOO are only slightly increased, but they are significantly higher for oxygen inside longer chains. Oxygen removal from within the chain requires not only additional high-barrier steps but also other reaction steps, e.g., formation of HCOO and OH from OCH_2_O, which are associated with higher activation barriers for oxygen inside chains. For a stepped surface where most oxygen is likely to be bound in chains, lower overoxidation rates are expected due to higher barriers for this oxygen type, which agrees with experimental results. For CO_2_ formation from formate, the barrier for the long chain is highest, in agreement with the observed formate stability in the presence of accumulated oxygen at 230 K.^16^ However, this does not yet explain why overoxidation on flat Au(111) is not similarly reduced. To address this, we performed AIMD simulations for oxygen adsorbed on both stepped Au(221) and flat Au(111). On stepped Au(221), initially short and later long, extended Au–O chains are formed accompanied by a strong surface restructuring (Figure 4 and Figure S3), which can be considered a general feature, as similar results were reported for stepped Au(511) surfaces exhibiting (100) terraces.^34^ In contrast to that, the calculations on Au(111) show no preferential formation of Au–O chains but rather repulsive interactions between oxygen atoms and a lower mobility of both surface Au atoms and oxygen atoms as compared to the stepped surface (see Figure S4). Previous STM studies similarly reported no formation of extended Au–O chains for Au(111), but rather formation of disordered AuO_*x*_ clusters at low oxygen coverages.^12−14^ Note that cluster formation depended on the oxidation conditions and was connected to expulsion of Au atoms from the herringbone reconstruction, which was not implemented in the calculations. These results suggest that more oxygen is present as individual atoms on Au(111) compared to on Au(332) with extended Au–O chains. Moreover, for small, disordered AuO_*x*_ clusters on Au(111), a reactivity similar to oxygen at the end of chains is expected. Thus, the formation of extended Au–O chains limits the availability of oxygen species with overoxidation potential on stepped gold surfaces and suppresses overoxidation.

**Figure 4:**
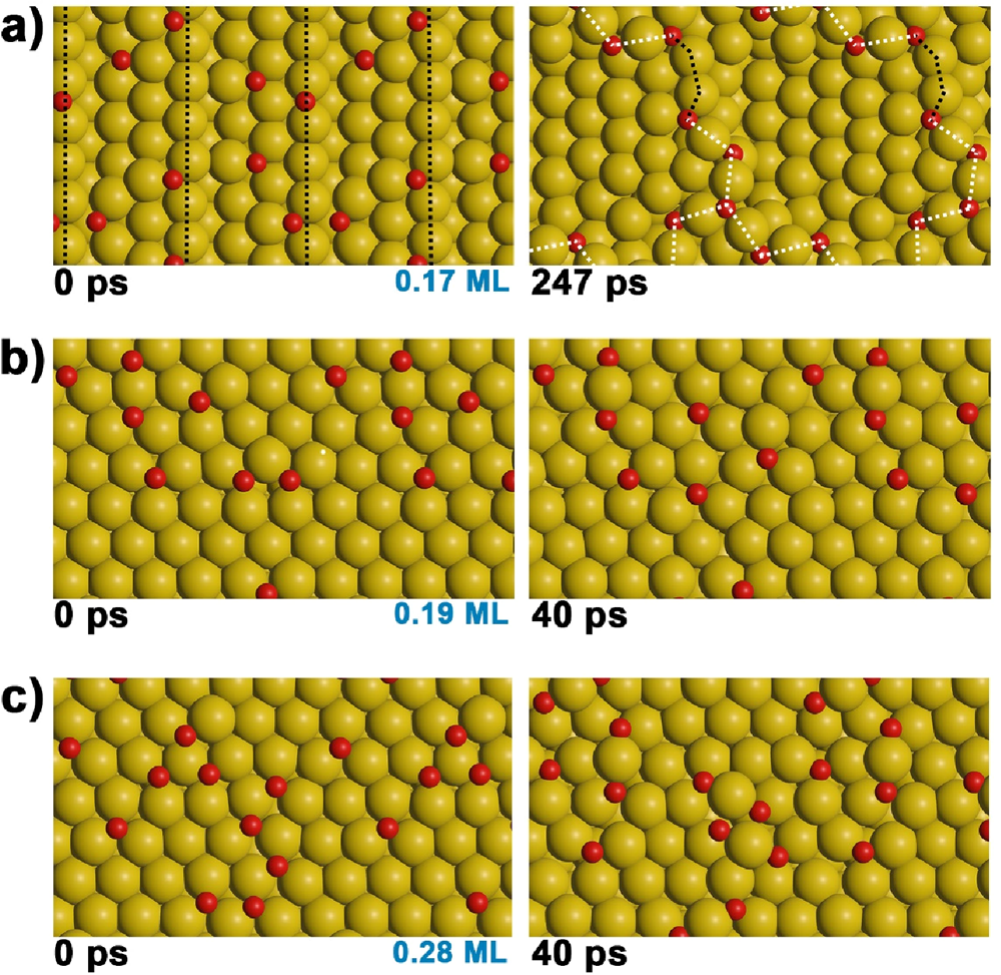
Snapshots from AIMD simulations of oxygen adsorbed on stepped Au(221) (a) and flat Au(111) (b and c). For Au(111), two different oxygen coverages, i.e., 0.19 ML (b) and 0.28 ML (c), are shown. Left, initial oxygen distribution; right, surface after evolution.

These findings have important implications: first, the observed preferential partial oxidation at low-coordinated sites is consistent with results for selective ethanol oxidation on supported gold nanoparticles,^35^ suggesting that their effects are not limited to methanol oxidation on npAu alone. In methanol oxidation, these sites have significantly different selectivities not only for partial oxidation to MeFo but also for overoxidation, with low-coordinated sites favoring the former and lowering the latter, as desired for an ideal partial oxidation catalyst. As noted above, higher local reactant concentrations at low-coordinated step sites promote the desired coupling reaction to MeFo. Furthermore, the preferential formation of extended Au–O chains at steps inhibits undesired overoxidation due to increased barriers for this pathway. The latter may also contribute to the understanding of the beneficial effect of ozone activation of npAu:^10^ as oxygen activation is the rate-limiting step in aerobic methanol oxidation on npAu,^7^ formation of extended Au–O chains at steps on nonactivated, presumably metallic npAu may be inhibited by the fast (nonselective) reaction of the generated oxygen atoms. In contrast, ozone-activated npAu is heavily oxygenated with increased Ag surface concentrations allowing for faster replenishment of activated oxygen to the reactive gold sites.^10^ Thus, after initial consumption of excess oxygen on terraces by methanol overoxidation,^10^ a steady state can be reached with sustained, extended Au–O chains allowing for high MeFo selectivity. A second beneficial effect of low-coordinated sites may be their preferential adsorption of reactants, which will increase their kinetic importance under low-coverage conditions, where low collision rates limit (coupling) reactions, as in the gas-phase methanol oxidation on npAu, as evidenced by a shift in selectivity from MeFo to formaldehyde for short contact times.^8^ Fast formation of Au–O chains at steps lowers the (atomic) oxygen concentration on terraces and thereby the associated reaction rates. Additionally, reactants, like methanol or formaldehyde, desorb faster from terraces than from more strongly binding steps, locally increasing the transient concentrations and thus the reaction rates at steps under multicollision conditions. This further shifts the product distribution toward the desired coupling and away from overoxidation. The adverse effect of (111) terraces on the MeFo selectivity is thus also mitigated, thereby improving the performance of the npAu catalysts.

In summary, we performed isothermal MB experiments under well-defined conditions and found that the (111) and stepped (332) surfaces show significantly different selectivities for methanol partial oxidation on gold. Thus, low-coordinated sites enhance the MeFo selectivity. Based on DFT calculations, the slower overoxidation at low-coordinated steps can be understood by the formation of extended Au–O chains, which lowers the probability for overoxidation. Our results highlight the importance of surface defects in facilitating the adsorbate-induced surface restructuring and self-organization of adsorbed atomic oxygen. These insights help us understand the high selectivity of npAu catalysts in the partial oxidation of alcohols.

## Data Availability

Raw and meta data is available under DOI: 10.5281/zenodo.11073692.

## Supporting Information Available

Experimental details, computational methods, IRAS spectra of formate accumulation, and integrated IRAS intensity of formate decomposition on Au(332) and Au(111) (PDF)
