# Supplementary material for: Partial Oxidation of Methanol on Gold: How Selectivity Is Steered by Low-Coordinated Sites
Source: ACS Catal. 2024 May 6;14(10):7901–6. doi: 10.1021/acscatal.3c04578 (PMC11106737; doi:10.1021/acscatal.3c04578)
Supplement: Supplementary file 1 — cs3c04578_si_001.pdf [file cs3c04578_si_001.pdf]

Supporting Information

Partial Oxidation of Methanol on Gold: How Selectivity is Steered by Low-Coordinated Sites

Salma Eltayeb,[a] Lenard L. Carroll,[b] Lukas Dippel,[a] Mersad Mostaghimi,[b,c] Wiebke Riedel,[a] Lyudmila V. Moskaleva,*[b] Thomas Risse*[a]

[a] Institut für Chemie und Biochemie, Freie Universität Berlin, Arnimallee 22, 14195 Berlin, Germany.
[b] Department of Chemistry, Faculty of Natural and Agricultural Sciences, University of the Free State, P.O. Box 339, Bloemfontein 9300, South Africa.
[c] Institute of Nanotechnology, Karlsruhe Institute of Technology (KIT), P.O. Box 3640, 76021 Karlsruhe, Germany.

E-mail: lyudmila.moskaleva@gmail.com, risse@chemie.fu-berlin.de

1. Experimental Details

The experiments were conducted in an ultra-high vacuum (UHV) machine which has been described in detail before.[1] In brief, the preparation chamber of the UHV apparatus is equipped a sputter gun (IQE 11/35, SPECS) for sample cleaning, a low energy electron diffraction (LEED) system (Omicron MCP LEED) for testing of the sample surface ordering, and a quadrupole mass spectrometer (Prisma, Pfeiffer) for temperature programmed desorption (TPD) measurements equipped with a Feulner cup to enhance sensitivity. In the main chamber, effusive molecular beams (MB)[2] are employed to expose the surface with methanol (or methyl formate), while a thermal atomic oxygen source (Dr Eberl MBE Komponenten GmbH) is used to supply atomic oxygen. These sources can be modulated by automated valves and shutters. A stagnation flow monitor with a high precision ion gauge (360 Stabil-Ion, Granville-Phillips) is used to measure the pressure and its spatial distribution at the sample position. The temporal evolution of gas phase species during pulsed MB experiments is followed by a quadrupole mass spectrometer (MAX-500HT, Extrel). In specific, the formation of methyl formate was monitored on its molecular ion signal (H_3COCHO^+ at m/z = 60). The methyl formate formation in the methanol oxidation was quantified by reference measurements applying well-defined fluxes of methyl formate provided by the effusive beams to the chamber.[3] Please note that direct MS detection of small amounts of CO_2 or formaldehyde in the gas phase is hindered by strong background and fragmentation signals. It should be emphasized that a delineation strategy to deconvolute the formaldehyde from methanol signals, as often used in TPR experiments, cannot be applied for the pulsed, isothermal MB experiments, as methanol is continuously applied at a large excess in the gas phase with respect to the oxygen atoms masking the correspondingly smaller formaldehyde contribution on e.g. m/z = 30 (molecular ion of formaldehyde). To monitor surface adsorbed species during the pulsed MB experiments, in situ IRAS measurements were conducted in grazing reflection geometry applying an IR spectrometer (IFS 66v, Bruker, 256 scans, nominal resolution of 4 cm^{-1}, zero filling factor of 16). The Au single crystals (10 mm diameter, MaTeck) are mounted by Mo-clamps onto a boron nitride heater (HT-01, Momentive) which is attached to a home-built Mo sample holder connected to a liquid nitrogen cooled Cu-block. A type K thermocouple inserted into a 0.2 mm hole in the Au crystal edge is employed to measure the sample temperature. To monitor the thermocouple voltage and control the sample temperature in TPD and isothermal experiments, a commercial PID controller (3508, Eurotherm) is used.

The Au samples were cleaned until sharp LEED images[4, 5] were observed by repeated cycles of Ar^+ ion sputtering (1000 V, 6–7 μA, 15 min for Au(332) and 30 min for Au(111)) and subsequent annealing i.vac. (for Au(332) to 1000 K for 10 min; for Au(111) to 900 K for 10 min and at 700 K for 30 min). Methanol (Honeywell Riedel de Haën, Chromasolv, ≥99.9 %) dried over molecular sieves (3 Å) and

further purified by repeated freeze-pump-thaw cycles was dosed onto the surface using an effusive molecular beam for which the pressure at the sample position was calibrated by the stagnation flow monitor as a function of the inlet pressure using Ar-gas. A thermal cracker (T = 1620 °C, 14.15 V, 15.52 A) and O_2 gas (99.998 %, Air Liquide) were employed to provide atomic and, thus, activated oxygen to the sample, as molecular oxygen does not dissociate under UHV-conditions on the gold surfaces.[6, 7] The atomic oxygen flux was calibrated by O_2-TPD measurements from Au(332) in comparison to O_2-TPD measurements from Pt(111) precovered with oxygen at 300 K exhibiting a p(2×2) structure with a coverage of 0.25 monolayers (ML).[8, 9] For both Au surfaces a saturation coverage of approx. 2 ML was found (1 ML corresponding to 1.4×10^{15} cm^{-2}, and thus, to one O atom per Au surface atom). The isothermal, pulsed molecular beam experiments were conducted with a constant methanol flux (4.3×10^{13} $s^{-1}cm^{-2}$), while atomic oxygen was pulsed (atomic oxygen flux: 0.4×10^{13} s^{-1} cm^{-2}, pulse length: 200 s, pulse delay: 300 s). Please note that an excess of methanol in the gas phase was provided as compared to atomic oxygen. Based on the quantified methyl formate formation rate, the methyl formate selectivity was accordingly calculated with respect to the provided flux of atomic oxygen considering that two oxygen atoms are required for methyl formate formation.

2. Computational Details

"Static" DFT computations of reaction energy profiles. The Au(221) surface was modelled with a p(4 ×1) unit cell in the lateral directions with a slab thickness of 7.7 Å and 7.2 Å vacuum space separating the slab from its periodic image in the Z direction. The unit cell parameters were a = 8.78 Å, b = 11.71 Å, c = 14.90 Å, and $\alpha = \beta = \gamma = 90°$. The atoms of the bottom half of the slab were frozen at their bulk positions while the remaining atoms were allowed to relax without constraints. The bulk gold positions of the bottom layers were taken from the calculated lattice constant of 4.17 Å. The Au–O chains were constructed along the steps of the surface. A "short" chain consisted of two O atoms connected in a linear O–Au–O fragment and a "long" chain consisted of three O atoms forming an O–Au–O–Au–O sequence and with two linear O–Au–O fragments, as shown in Figure 3a of the main text.

The "static" DFT calculations in this study were carried out with Vienna ab initio simulation package (VASP)[7, 8] using the projector augmented wave (PAW)[9, 10] method and a plane wave basis set. Perdew, Burke, and Ernzerhof (PBE)[11, 12] exchange-correlation functional was applied in the calculations. A 5 × 5 × 1 k-point mesh, and the k-point sampling scheme of Monkhorst-Pack[13] grids for the integrations within the Brillouin zone were used in the calculations. The calculations were performed with a plane-wave cutoff energy of 415 eV and a larger kinetic energy cutoff of 645 eV. In the calculations, we used Methfessel-Paxton order 1 smearing scheme[14] with a smearing parameter of 0.05 eV. The relaxation of the surfaces (with the conjugate gradient method) was performed until the force acting on each atom was less than 0.02 eV/Å, while the energy was converged with a tolerance of 10^{-6} eV. The minimum energy reaction paths were determined by the climbing-image nudged elastic band method (ci-NEB)[15], an improved method with respect to the conventional nudged elastic band method (NEB)[16] and the transition state structure searches were further refined by the dimer[17] method.

Ab Initio Molecular Dynamics. AIMD simulations were executed using the CP2K computational code, renowned for its computational efficiency.[18] CP2K was used for both the optimization of initial structures and the ensuing AIMD simulations. In the case of the Au(221) structure, a p(2×6) unit cell was adopted, featuring a slab thickness of approximately 8 Å. A vacuum region of around 10 Å was incorporated to separate the slab from its periodic counterpart along the Z direction. While the lower layers of the slab remained constrained, the upper layers were permitted to relax freely. For the Au(111) surface, characterized by a p(6×6) unit cell (36 Au atoms per layer), a comparable approach was employed. Here, the bottom two layers were held in a fixed position, while the remaining upper layers underwent relaxation. The slab thickness was set at approximately 9 Å, accompanied by a vacuum

region of equivalent dimensions. Oxygen atoms were placed pseudo-randomly on the Au(111) and Au(221) surfaces using an in-house python script that makes use of a convex hull algorithm, triangulation, point generation and distance checks. In both scenarios, simulations were conducted in the NVT ensemble, and the Nosé-Hoover thermostat was selected as the temperature control mechanism. These choices were based on prior research within our group and established practices. A time step of 1 fs was selected for the simulations, with snapshots recorded every 20 fs. Exchange-correlation energy calculations relied on the Perdew, Burke, and Ernzerhof (PBE) functional.[11,12] The Goedecker-Teter-Hutter, Perdew, Burke, and Ernzerhof (GTH-PBE) pseudopotential,[19] coupled with Gaussian and plane-wave (GPW) basis sets,[20] was used alongside a multigrid cutoff energy of 500 Rydberg. To mitigate basis set superposition errors, CP2K's double-zeta basis set optimized for GTH pseudopotentials, suitable for both solid and molecular computations, was implemented.[21] To reduce computational overhead, Brillouin zone integration was confined to the Γ point for AIMD simulations. Dispersion corrections were omitted to economize computational resources. The convergence threshold for self-consistent electronic minimization was set at 10^{-6} eV. To efficiently explore a broad configuration space of surface arrangements, a statistical sampling approach was adopted at an elevated temperature of 700 K. This strategy has previously demonstrated success in a variety of AIMD studies, for example in a related study from 2017.[22]

In Figure S3, the snapshots of these AIMD simulations are compared at 0 ps and 25 ps, while in Figure S4 the root mean squared displacement (RMSD) of the gold surfaces and the oxygen atoms adsorbed atop them are compared. This allows us to quantify, how the surface Au atoms and O atoms displace over time which can be used as a measure of mobility. From Figure S4, we can infer that the surface Au atoms on Au(221) are significantly more mobile than those of Au(111). For Au(111), the higher O coverage leads to a larger Au displacement than the lower O coverage. The O atoms on Au(221) also end up being significantly more mobile than those on Au(111), at times by a factor of 4. Similar to the displacement of the Au surface atoms, the higher O coverage on Au(111) leads also to an increased displacement of the O atoms than a lower O coverage. The RMSD values were calculated using:

$$\mathrm{RMSD}(t) = \frac{\sum_{n=1}^{N} \sqrt{\left(x_n(t) - x_n(0)\right)^2 + \left(y_n(t) - y_n(0)\right)^2 + \left(z_n(t) - z_n(0)\right)^2}}{N}$$

where $x_n(t)$ is the x-position of an atom n at time t, $x_n(0)$ is the x-position of an atom n at the initial point, and so forth.

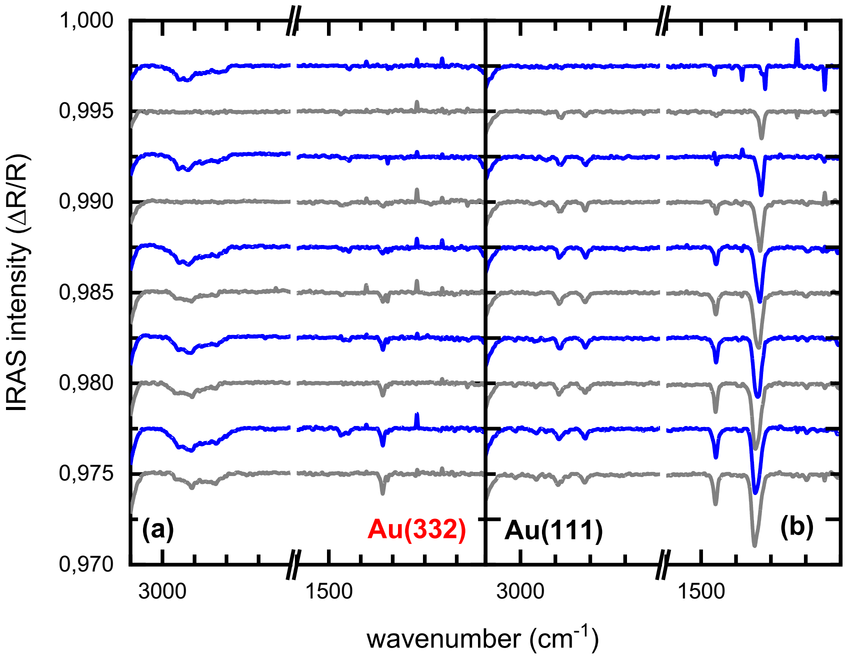

Figure S1 IRAS spectra measured across the pulse sequence of MB experiments at 230 K exposing (a) Au(332) and (b) Au(111) continuously with methanol (flux of 4.3×10^{13} s^{-1} cm^{-2}), while pulsing atomic oxygen (flux of 0.4×10^{13} s^{-1} cm^{-2}, 200 s on, 300 s off). Spectra were measured from the first (top) to the fifth (bottom) oxygen pulse, both during the pulse (blue) and in the delay time between pulses (grey). Based on literature reports, the signals around 1330 cm^{-1}, 2815 cm^{-1} and 2890 cm^{-1} are assigned to the υ_s(OCO), a combination of υ_s(CH)+υ_a(OCO) and υ_a(CH) of bidentate formate species.[23-24] The signal at 1460 cm^{-1}, which is not clearly detected for Au(332), has not been reported for formate species on Au(111) before[23] hindering an assignment of this signal. However, it is in the range of (blue-shifted) δ(CH) or δ(OH) of formic acid.[25-26] For Au(111), the intensity of signals associated with formate species increases markedly across the pulse sequence attesting to significant formate accumulation, while the intensity increase on Au(332) is significantly smaller. In specific, the integrated intensity of the signal around 1330 cm^{-1} is a factor of approx. 8 smaller on Au(332) as compared to Au(111) suggesting a significantly smaller amount of formate on Au(332). It should be noted that the precise adsorption geometry of the formate species may differ for the two surfaces. As the IRAS intensity depends on the transition dipole moment perpendicular to the surface, changes in the adsorption geometry will also affect the signal intensity. Yet, the strong difference in signal intensity for Au(332) and Au(111) cannot be explained by a small tilting of the formate with respect to the surface normal, but would require an almost parallel orientation of the OCO-bonds of formate to the surface plane for Au(332). Thus, the difference in signal intensity can be attributed (at least partially) to changes in the amount of formate species.

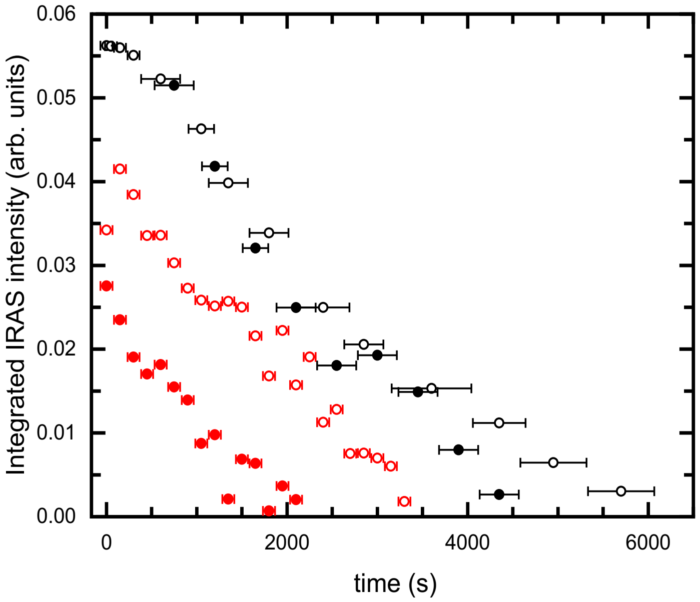

Figure S2 Formate decomposition by exposure to atomic oxygen (flux of 0.08×10^{13} s^{-1} cm^{-2}) at 230 K (filled symbols) and 210 K (open symbols) for formate-precovered Au(332) (red) and Au(111) (black). The IRAS intensity was integrated in the range of the formate signal attributed to υ_s(OCO)-vibration, in specific, between 1319 cm^{-1} and 1355 cm^{-1}. It can be seen that the formate decomposition rate, *i.e.* the slope, is similar for both surfaces and overall slow. Please note that this reactivity in the isothermal pulsed MB experiments differs from TPR experiments with accumulated AuO_x-phases which allow for formate decomposition at higher temperatures.[10, 11]

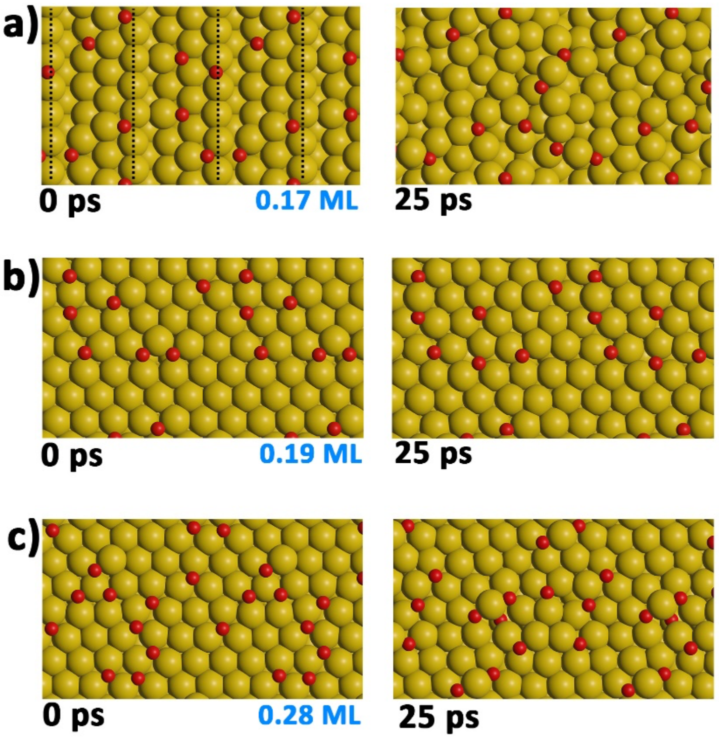

Figure S3 Snapshots from AIMD simulations illustrating different degree of surface restructuring induced by adsorbed atomic oxygen on stepped Au(221) (a) and flat Au(111) (b,c). For Au(111), two different oxygen coverages, *i.e.* 0.19 ML (b) and 0.28 ML (c), are shown; left: initial oxygen distribution; right: surface after 25 ps evolution.

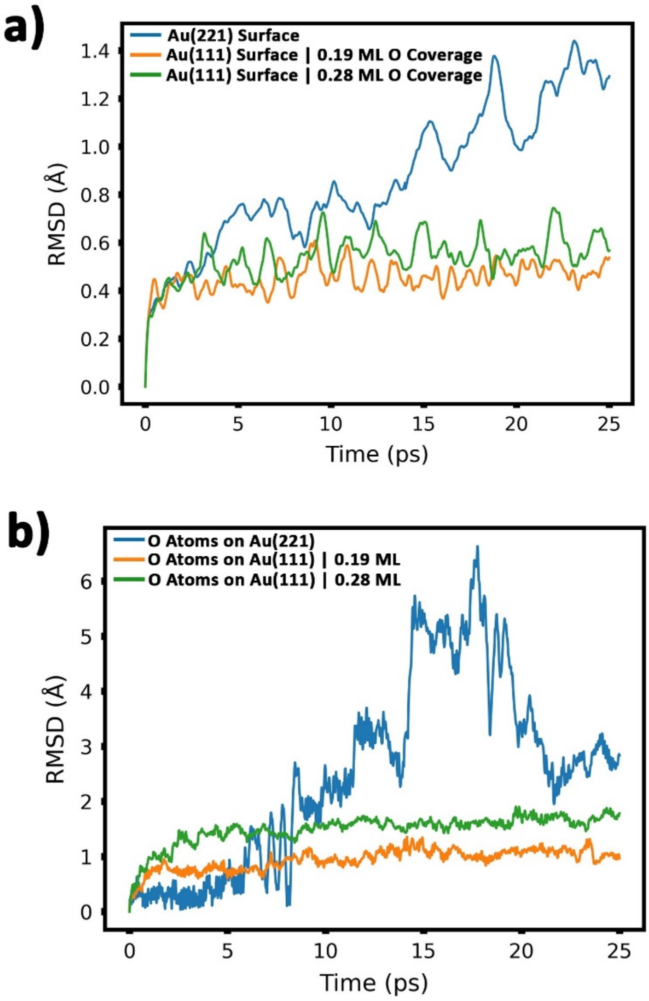

Figure S4 RMSD plots comparing (a) the displacement of the Au(111) and Au(221) surface Au atoms; (b) the displacement of the oxygen atoms on the Au(111) and Au(221) surfaces.

References

1. Moreira, R. Setup of a Molecular Beam Apparatus to Study the Reactivity of Single Crystal Surfaces and its Application to CO Oxidation on Au(332). PhD Thesis, Freie Universtität Berlin, Berlin, 2018.
2. Libuda, J.; Meusel, I.; Hartmann, J.; Freund, H. J., A molecular beam/surface spectroscopy apparatus for the study of reactions on complex model catalysts. *Rev. Sci. Instrum.* **2000,** *71* (12), 4395-4408.
3. Feldt, C. D.; Kirschbaum, T.; Low, J. L.; Riedel, W.; Risse, T., Methanol oxidation on Au(332): Methyl formate selectivity and surface deactivation under isothermal conditions. *Catal. Sci. Technol.* **2021,** *12* (5), 1418-1428.
4. Prieto, M. J.; Carbonio, E. A.; Landers, R.; de Siervo, A., Structural and Electronic Characterization of Co nanostructures on Au(332). *Surf. Sci.* **2013,** *617*, 87-93.
5. van Hove, M. A.; Koestner, R. J.; Stair, P. C.; Biberian, J. P.; Kesmodel, L. L.; Bartos, I.; Somorjai, G. A., The surface reconstructions of the (100) crystal faces of iridium, platinum and gold. 1. Experimental observations and possible structural models. *Surf. Sci.* **1981,** *103* (1), 189-217.
6. Pireaux, J. J.; Chtaib, M.; Delrue, J. P.; Thiry, P. A.; Liehr, M.; Caudano, R., Electron Spectroscopic Characterization of Oxygen-Adsorption on Gold Surfaces. 1. Substrate Impurity Effects on Molecular Oxygen Adsorption in Ultra High-Vacuum. *Surf. Sci.* **1984,** *141* (1), 211-220.

7. Sault, A. G.; Madix, R. J.; Campbell, C. T., Adsorption of Oxygen and Hydrogen on Au(110)-(1x2). *Surf. Sci.* **1986,** *169* (2-3), 347-356.
8. Mortensen, K.; Klink, C.; Jensen, F.; Besenbacher, F.; Stensgaard, I., Adsorption position of oxygen on the Pt(111) surface. *Surf. Sci.* **1989,** *220* (2-3), L701-L708.
9. Norton, P. R.; Davies, J. A.; Jackman, T. E., Absolute coverages of CO and O on Pt(111) - comparison of saturation CO coverages on Pt(100), (110) and (111) surfaces *Surf. Sci.* **1982,** *122* (1), L593-L600.
10. Xu, B. J.; Liu, X. Y.; Haubrich, J.; Madix, R. J.; Friend, C. M., Selectivity Control in Gold-Mediated Esterification of Methanol. *Angew. Chem. Int. Ed.* **2009,** *48* (23), 4206-4209.
11. Wu, Z. F.; Jiang, Z. Q.; Jin, Y. K.; Xiong, F.; Sun, G. H.; Huang, W. X., Oxidation of formic acid on stepped Au(997) surface. *Chin. J. Catal.* **2016,** *37* (10), 1738-1746.
